# Chronic Pica Resulting in Perforative Peritonitis: A Case Report

**DOI:** 10.7759/cureus.109020

**Published:** 2026-05-17

**Authors:** Rohan P Doshi, Harsh J Barot, Nitin Borle, Sachin Suryawanshi

**Affiliations:** 1 Surgery, Topiwala National Medical College and Bai Yamunabai Laxman Nair Charitable Hospital, Mumbai, IND; 2 General Surgery, Topiwala National Medical College and Bai Yamunabai Laxman Nair Charitable Hospital, Mumbai, IND; 3 General Surgery, Seth GS Medical College (GSMC) and King Edward Memorial (KEM) Hospital, Mumbai, IND

**Keywords:** foreign bodies, intellectually disabled persons, intestinal obstruction, intestinal perforation, laparotomy, perforative peritonitis, pica, postoperative complications, sepsis, small bowel obstruction

## Abstract

Foreign body (FB) ingestion is a frequent clinical presentation, particularly among children and individuals with intellectual disabilities. However, recurrent ingestion associated with pica represents a high-risk subset, often leading to cumulative gastrointestinal injury, operative complexity, and increased mortality. We report the case of a 23-year-old male patient with severe intellectual disability who presented with abdominal pain and distension of five-day duration. The patient had a significant history of recurrent FB ingestion, predominantly metallic objects, and his status was post prior surgical intervention. One year prior, he had undergone emergency laparotomy for cecal perforation secondary to ingestion of a metallic FB. On current evaluation, imaging revealed features of small bowel obstruction with suspected perforative peritonitis and multiple radio-opaque FBs. Exploratory laparotomy demonstrated extensive intra-abdominal pathology, including 12 sealed small bowel perforations, enteroenteric fistulae, and dense adhesions involving the small bowel, transverse colon, and stomach. Numerous FBs were identified, including batteries, screws, bolts, nuts, zippers, wooden sticks, and hair. A 60 cm segment of diseased small bowel was resected, and a double-barrel jejuno-ileostomy was performed in view of severe contamination and hemodynamic instability. Postoperatively, the patient required mechanical ventilation and inotropic support, but developed rapidly progressive septic shock leading to multi-organ dysfunction syndrome (MODS). The patient expired on postoperative day one. This case illustrates the pathophysiological progression of chronic pica, where recurrent ingestion leads to cumulative bowel injury, adhesions, fistula formation, and multiple perforations. It highlights the significant surgical challenges and poor prognostic factors, including delayed presentation, prior surgery, and severe intra-abdominal contamination. Notably, the case underscores a critical preventive failure, as the absence of sustained psychiatric intervention allowed recurrence and progression to fatal complications. Severe, recurrent pica should be recognized as a high-risk condition with potential for catastrophic gastrointestinal injury and mortality. Early identification, structured psychiatric follow-up, and a multidisciplinary preventive approach are essential to reduce recurrence and improve outcomes.

## Introduction

Foreign body (FB) ingestion is a common clinical presentation, particularly among children and individuals with neuropsychiatric disorders. While most ingested objects pass spontaneously through the gastrointestinal (GI) tract, approximately 10-20% require endoscopic retrieval and fewer than 1% necessitate surgical intervention due to complications such as obstruction, perforation, or fistula formation [1]. Among predisposing conditions, pica, defined in the Diagnostic and Statistical Manual of Mental Disorders, Fifth Edition (DSM-5) as the persistent ingestion of non-nutritive substances, is frequently associated with intellectual disability and contributes to recurrent and high-risk ingestion behaviors [2].

Although isolated FB ingestion is well described, severe recurrent pica resulting in repeated ingestion of sharp and bulky objects with cumulative multi-segment bowel injury remains rarely reported, particularly in adults with intellectual disability [2,3]. This creates significant diagnostic and therapeutic challenges, including delayed presentation, cumulative bowel damage, and increased operative complexity.

The clinical problem addressed in this report is the management of recurrent, high-risk FB ingestion resulting in complex small bowel pathology, where both surgical decision-making and long-term prevention strategies are critical.

We present a case of recurrent FB ingestion in the setting of pica, culminating in severe small bowel complications, to highlight the operative challenges, risk of morbidity and mortality, and the essential role of integrated psychiatric intervention in preventing recurrence.

## Case presentation

This case report involves a 23-year-old male patient with a known history of intellectual disability, characterized by marked impairment in adaptive functioning across conceptual, social, and practical domains. He was nonverbal with minimal communicative ability, demonstrated limited understanding of environmental risks, and was dependent on caregivers for activities of daily living. The patient had a history of pica, with recurrent ingestion of non-nutritive and potentially harmful objects. He presented with abdominal pain, distention, and features suggestive of intestinal obstruction. He had undergone surgery one year prior for a cecal perforation caused by the ingestion of a nail. Initially, he was admitted to a private hospital with similar complaints, where imaging and investigations were conducted, and surgical intervention was advised. The patient was subsequently referred to our institution for further management.

On admission, the patient was tachycardic and borderline hypotensive, with a pulse rate of 116 beats/min (reference range: 60-100 beats/min), blood pressure of 100/60 mmHg (reference systolic blood pressure: 90-120 mmHg), respiratory rate of 22 breaths/min (reference range: 12-20 breaths/min), temperature of 37.8°C (reference range: 36.1-37.2°C), and oxygen saturation of 97% on room air (reference value: ≥95%). Clinically, he appeared ill and dehydrated, raising concern for early systemic inflammatory response. From a hemodynamic perspective, he was hemodynamically borderline with compensated shock physiology, characterized by tachycardia with relative hypotension but preserved oxygenation. Serum lactate was not available; however, there were no overt clinical signs of hypoperfusion.

Table 1 shows the patient's complete blood count values.

**Table 1:** Complete blood count at the time of admission

Parameter	Value	Reference range
Hemoglobin	9.2 g/dL	13-17 g/dL
Hematocrit	30.20%	40-55%
White blood cell count	14,400/cu.mm	4,000- 10,000/cu.mm
Platelet	2,70,000/μL	1,50,000 -4,00,000/μL

**Figure 1:**
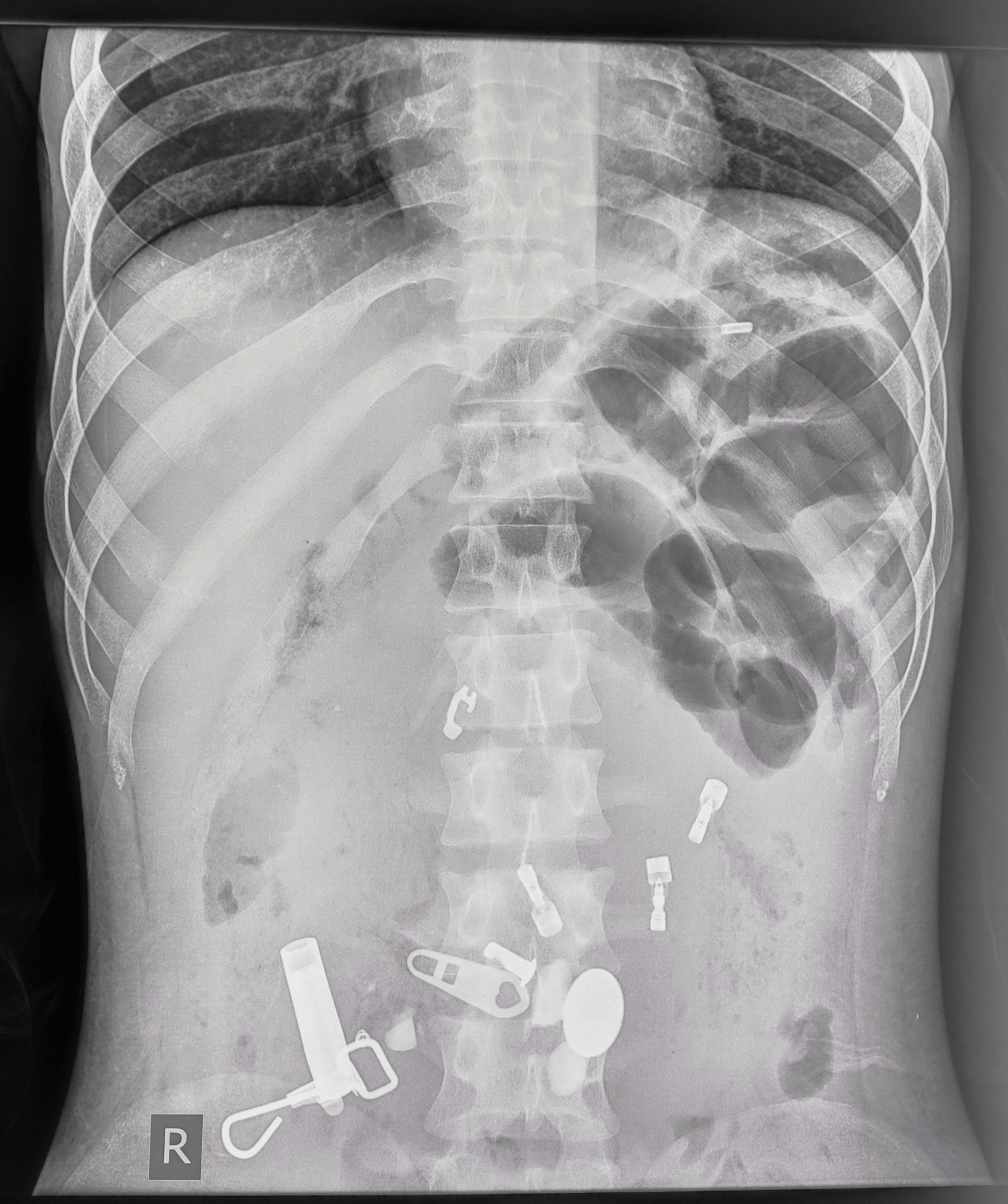
X-ray of the abdomen showing foreign bodies in the gastrointestinal tract

An erect abdominal radiograph revealed multiple air-fluid levels, indicative of small bowel obstruction, with numerous radio-opaque densities consistent with ingested metallic FBs scattered throughout the small bowel and colon (Figure 1).

**Figure 2:**
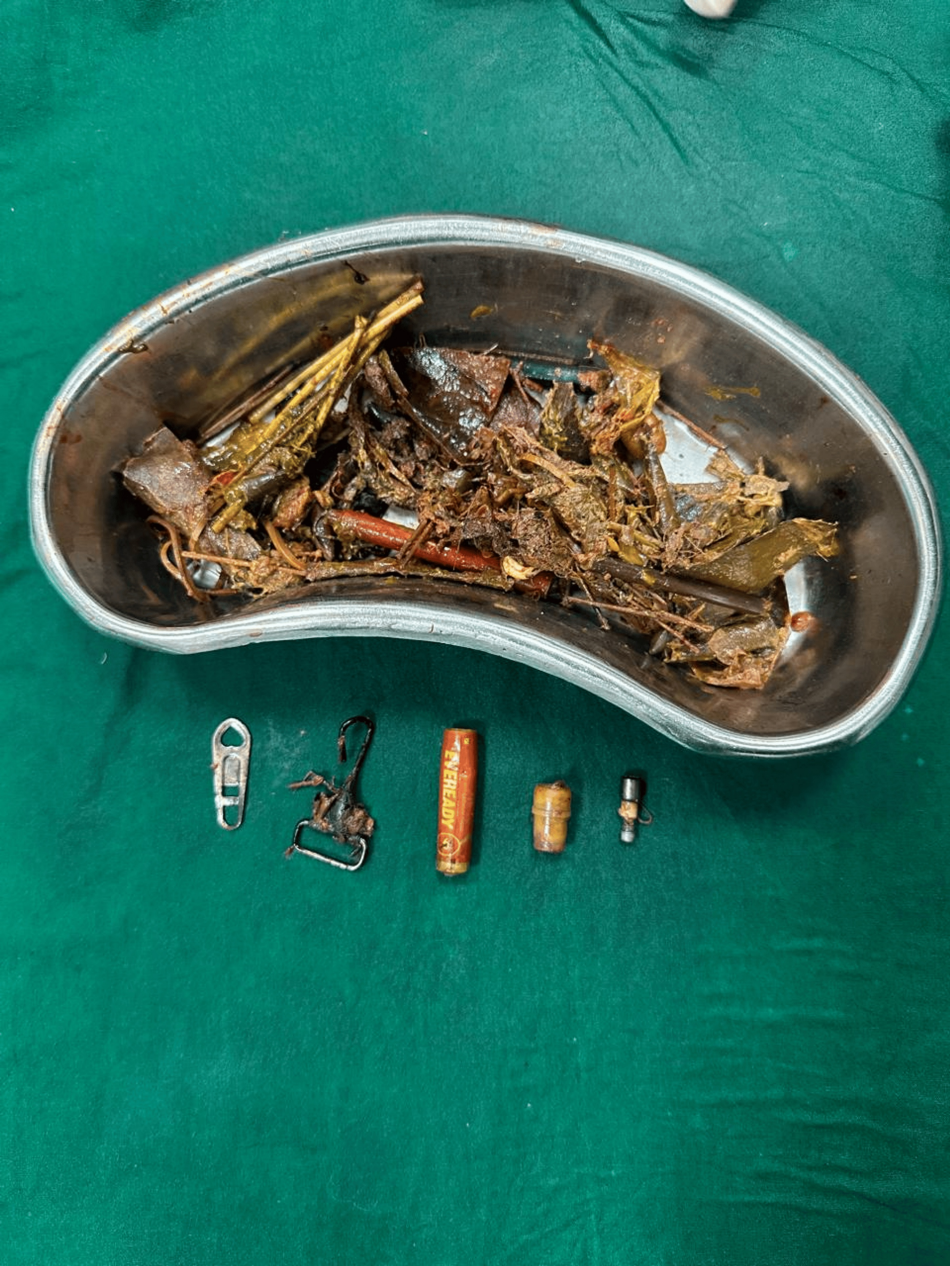
Ingested foreign bodies

**Figure 3:**
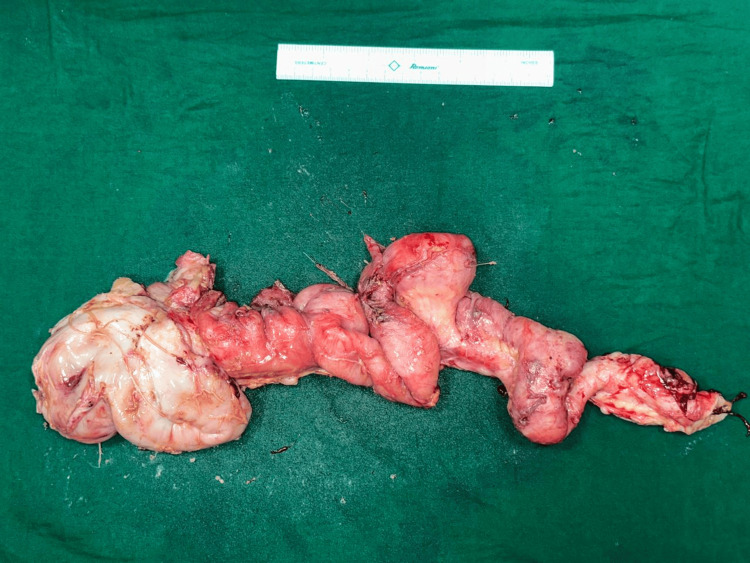
Specimen

A decision for exploratory laparotomy was taken after resuscitation of patient. Approximately 300 ml of feculent contamination was noted. Intraoperative findings revealed 12 sealed small bowel perforations with associated enteroenteric fistula formation, along with dense interloop adhesions involving the small bowel, transverse colon, and stomach. Multiple FBs, comprising metallic and organic materials, were present throughout the gastrointestinal tract (Figures 2, 3).

**Figure 4:**
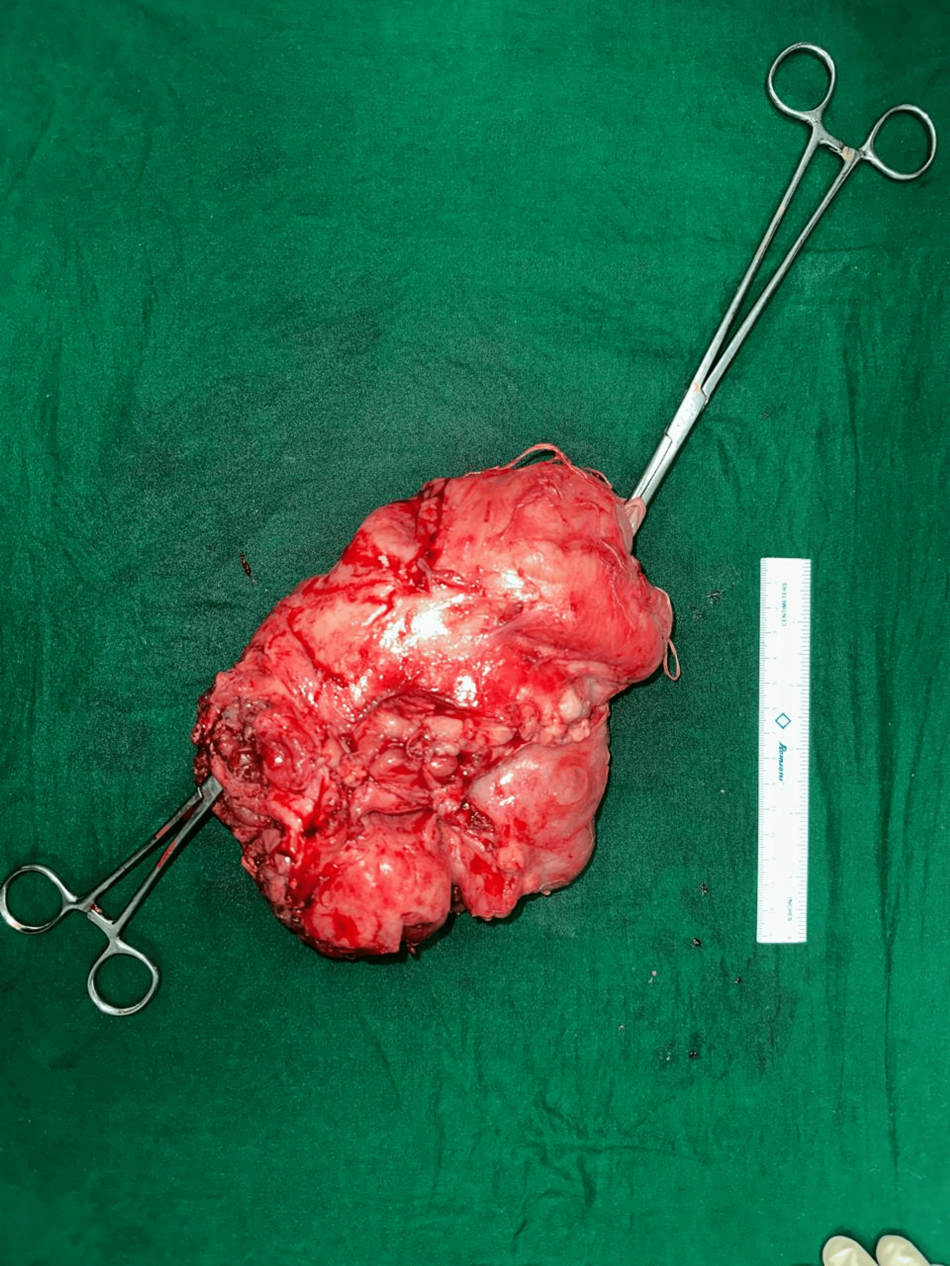
Resected specimen consisting of approximately 60 cm of jejunum with dense adhesions, multiple sealed perforations, and enteroenteric fistulae

Intraoperatively, the small bowel demonstrated multiple sealed perforations with dense interloop adhesions and gross feculent contamination. Approximately 60 cm of diseased small bowel was resected (Figure 4), encompassing segments with compromised viability and multiple perforations.

The degree of intra-abdominal contamination was severe (Hinchey stage IV) [4]. Given the presence of feculent peritonitis, extensive adhesions, and the patient’s hemodynamic instability, primary anastomosis was considered high risk. Therefore, a double-barrel stoma was fashioned to reduce the risk of anastomotic failure and facilitate postoperative management.

Postoperatively, the patient was admitted to the intensive care unit and required mechanical ventilatory support and inotropic support for persistent hemodynamic instability. Despite aggressive resuscitative measures, including broad-spectrum antibiotics and organ support, the patient developed rapidly progressive septic shock leading to multi-organ dysfunction syndrome (MODS).

The clinical condition deteriorated despite maximal supportive care, and the patient succumbed on postoperative day one, with the cause of death attributed to septic shock secondary to extensive intra-abdominal sepsis.

## Discussion

Pica is commonly observed in individuals with intellectual disabilities and involves the compulsive ingestion of non-food items, often leading to significant gastrointestinal complications [2]. While most literature describes isolated or episodic ingestion, chronic and recurrent ingestion represents a distinct and more hazardous clinical entity, characterized by cumulative gastrointestinal injury over time.

Pathophysiological progression in chronic pica follows a stepwise pattern: repeated ingestion of heterogeneous FBs leads to chronic mucosal trauma and localized inflammation, progressing to fibrosis and dense adhesions. These adhesions predispose to altered bowel motility, luminal narrowing, and intermittent obstruction, eventually culminating in pressure necrosis, sealed or free perforations, and fistula formation [3,5]. The present case exemplifies this continuum, with multiple sealed perforations, enteroenteric fistulae, and extensive intra-abdominal adhesions, reflecting long-standing, repetitive injury rather than an acute event.

Diagnostic and surgical challenges in such cases are substantial. Although imaging, particularly computed tomography (CT), plays a critical role in identifying FBs and associated complications, the true extent of bowel damage is often underestimated preoperatively. Chronic inflammation and adhesions obscure anatomical planes, making operative dissection difficult and increasing the risk of inadvertent enterotomies. These cases are frequently associated with recurrent presentations and substantial operative complexity, often requiring individualized intraoperative decision-making rather than a standardized surgical approach [3,5]. While many ingested FBs pass harmlessly through the gastrointestinal tract, sharp or bulky objects pose a higher risk of obstruction, perforation, and fistulization [6]. In the present case, the presence of multiple perforations, dense adhesions, and feculent contamination (Hinchey IV) necessitated bowel resection and stoma formation, with avoidance of primary anastomosis due to the high risk of failure.

Determinants of poor outcomes in such patients are multifactorial. Key contributors include delayed presentation, chronicity of ingestion leading to cumulative bowel damage, severity of intra-abdominal contamination, and physiological compromise at presentation. Additionally, patients with intellectual disability often have limited ability to communicate symptoms, resulting in delayed diagnosis and advanced disease at the time of intervention. Prior subclinical episodes of perforation and healing may further exacerbate adhesion formation and operative difficulty [3,5,7]. In this case, the combination of extensive contamination, multiple perforations, and hemodynamic instability contributed to rapid progression to septic shock and multi-organ dysfunction syndrome, culminating in mortality despite surgical intervention.

Preventive failure represents a critical aspect of this case. Despite known high-risk behavior (pica), the absence of sustained psychiatric intervention and caregiver supervision likely permitted recurrent ingestion and progressive gastrointestinal injury. Prevention remains the cornerstone of management, as highlighted in prior studies [8]. Behavioral therapy, environmental control, and caregiver education are essential to reduce recurrence. A multidisciplinary approach, involving psychiatry, gastroenterology, surgery, and dedicated caregiver support, is necessary to address both the behavioral and medical dimensions of the disorder.

Existing literature on foreign body ingestion largely focuses on acute presentations. AlMuhsin et al. (2021) highlighted the complexity of managing massive bezoars and dense adhesions in similar clinical scenarios [3]. Salman et al. demonstrated that most ingested FB pass spontaneously, with a minority requiring intervention [9]. However, such data may underestimate the risk associated with chronic, repetitive ingestion, particularly in vulnerable populations. Unusual complications, such as appendiceal lodging of foreign bodies requiring surgical removal, have also been described [9]. Differences between intentional and accidental ingestion patterns further highlight the heterogeneity of this condition, with certain high-risk groups demonstrating recurrent behavior [10]. While FB ingestion in children is well documented, most commonly involving coins and other non-food items [7], adult presentations are more frequently associated with intentional or psychiatric conditions such as pica and tend to involve recurrent ingestion of heterogeneous and high-risk objects [2,7,10]. Consequently, adult cases are more likely to present with cumulative gastrointestinal injury, operative complexity, and poorer outcomes, as illustrated in the present case.

This case underscores a critical but under-recognized clinical message: severe, recurrent pica can lead to progressive, multi-segment gastrointestinal injury with high operative complexity and significant mortality risk. Early identification of high-risk individuals, timely psychiatric intervention, and close caregiver supervision are essential to prevent catastrophic outcomes. From a surgical perspective, clinicians should anticipate extensive adhesions, multiple occult perforations, and severe contamination, and plan operative strategies accordingly, often favoring damage-control approaches over definitive reconstruction in unstable patients.

## Conclusions

This case highlights the catastrophic consequences of recurrent FB ingestion in the setting of chronic pica, particularly when preventive strategies are inadequate. Unlike isolated ingestion events, repeated episodes lead to cumulative bowel injury, resulting in dense adhesions, multiple occult perforations, and complex fistulous disease, significantly increasing operative difficulty and mortality risk. Importantly, this case underscores the high risk of recurrence even after prior surgical intervention, as the underlying behavioral disorder remains unaddressed. Surgical management alone is therefore insufficient and may only provide temporary resolution while predisposing to further morbidity with subsequent episodes.

This case underscores the importance of long-term psychiatric management in patients with pica and recurrent foreign body ingestion. Structured behavioral therapy, environmental modification, and close caregiver supervision are essential to reduce recurrence and prevent progressive gastrointestinal injury. Chronic pica should be recognized as a high-risk condition requiring coordinated multidisciplinary care involving surgeons, psychiatrists, gastroenterologists, and caregivers to minimize morbidity and mortality.
